# Learning smoothing models of copy number profiles using breakpoint annotations

**DOI:** 10.1186/1471-2105-14-164

**Published:** 2013-05-22

**Authors:** Toby Dylan Hocking, Gudrun Schleiermacher, Isabelle Janoueix-Lerosey, Valentina Boeva, Julie Cappo, Olivier Delattre, Francis Bach, Jean-Philippe Vert

**Affiliations:** 1 INRIA Sierra project-team, Paris, F-75013, France; 2 Centre for computational biology, Mines ParisTech, Fontainebleau, F-77300, France; 3 Institut Curie, Paris, France; 4 INSERM U900, Paris, F-75248, France; 5 INSERM U830, Paris, F-75248, France

## Abstract

**Background:**

Many models have been proposed to detect copy number alterations in chromosomal copy number profiles, but it is usually not obvious to decide which is most effective for a given data set. Furthermore, most methods have a smoothing parameter that determines the number of breakpoints and must be chosen using various heuristics.

**Results:**

We present three contributions for copy number profile smoothing model selection. First, we propose to select the model and degree of smoothness that maximizes agreement with visual breakpoint region annotations. Second, we develop cross-validation procedures to estimate the error of the trained models. Third, we apply these methods to compare 17 smoothing models on a new database of 575 annotated neuroblastoma copy number profiles, which we make available as a public benchmark for testing new algorithms.

**Conclusions:**

Whereas previous studies have been qualitative or limited to simulated data, our annotation-guided approach is quantitative and suggests which algorithms are fastest and most accurate in practice on real data. In the neuroblastoma data, the equivalent pelt.n and cghseg.k methods were the best breakpoint detectors, and exhibited reasonable computation times.

## Background

### The need for smoothing model selection criteria

DNA copy number alterations (CNAs) can result from various types of genomic rearrangements, and are important in the study of many types of cancer [1]. In particular, clinical outcome of patients with neuroblastoma has been shown to be worse for tumors with segmental alterations or breakpoints in specific genomic regions [2,3]. Thus, to construct an accurate predictive model of clinical outcome for these tumors, we must first accurately detect the precise location of each breakpoint.

In recent years, array comparative genomic hybridization (aCGH) microarrays have been developed as genome-wide assays for CNAs, using the fact that microarray fluoresence intensity is proportional to DNA copy number [4]. In parallel, there have been many new mathematical models proposed to smooth the noisy signals from these microarray assays in order to recover the CNAs [5-12]. Each model has different assumptions about the data, and it is not obvious to decide which model is appropriate for a given data set.

Furthermore, most models have parameters that control the degree of smoothness. Varying these smoothing parameters will vary the number of detected breakpoints. Most authors give default values that accurately detect breakpoints on some data, but do not necessarily generalize well to other data. There are some specific criteria for choosing the degree of smoothness in some models [13-15], but it is impossible to verify whether or not the mathematical assumptions of these models are satisfied for real noisy microarray data.

To motivate the use of their cghFLasso smoothing model, Tibshirani and Wang write “The results of a CGH experiment are often interpreted by a biologist, but this is time consuming and not necessarily very accurate” [8].

In contrast, this paper takes the opposite view and assumes that the expert interpretation of the biologist is the gold standard which a model should attain. The first contribution of this paper is a smoothing model training protocol based on this assumption.

In practice, visualization tools such as VAMP are used to plot the normalized microarray signals against genomic position for interpretation by an expert biologist looking for CNAs [16]. Then the biologist plots a model and varies its smoothness parameter, until the model seems to capture all the visible breakpoints the data. In this article, we make this model training protocol concrete by using an annotation database to encode the expert’s interpretation.

**Figure 1:**
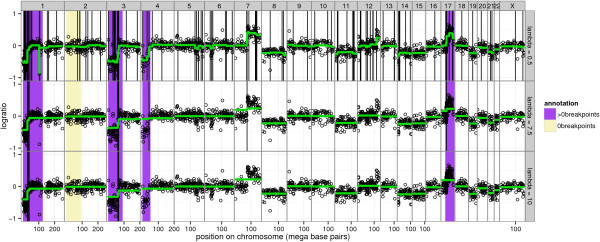
**Breakpoint annotations quantify the accuracy of a smoothing model.** Model agreement to annotated regions can be measured by examining the positions of predicted breakpoints ${\hat{b}}^{\lambda }$ (vertical black lines) observed in the smoothing model ${ŷ}^{\lambda }$ (green lines). Black circles show logratio measurements *y* plotted against position *p* for a single profile *i*=375. Chromosomes are shown in panels from left to right, and different values of the smoothing parameter *λ* in the flsa model are shown in panels from top to bottom. Models with too many breakpoints ( *λ*=0.5) and too few breakpoints ( *λ*=10) are suboptimal, so we select an intermediate model ( *λ*=7.5) that maximizes agreement with the annotations, thus detecting a new breakpoint on chromosome 7 which was not annotated.

**Figure 2:**
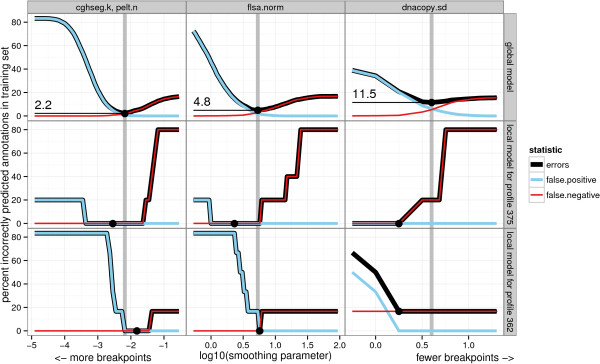
**Selecting the model that minimizes the breakpoint annotation error.** Training error functions for global and local models plotted against smoothing parameter *λ*. The original annotation data set was used to calculate the annotation error. In the top row panels, we plot *E*^global^(*λ*) from Equation 6, and in the other rows, we plot $E_{i}^{\text{local}}(\lambda )$ from Equation 5. Each column of plots shows the error of a particular algorithm, and the minimum chosen using the global training procedure is shown using a vertical grey line. Note that the local model training error can be reduced by moving from the globally optimal smoothing parameter $\hat{\lambda }$ to a local value ${\hat{\lambda }}_{i}$, as in profile *i*=375 for dnacopy.sd. For the local models trained on single profiles, many smoothing parameters attain the minimum. So we use the protocol described in the “Selecting the optimal degree of smoothness” section to select the best value, shown as a black dot.

The particular type of annotations that we propose are counts of breakpoints in genomic regions. By visual inspection of the noisy signal, it is not obvious to locate the exact location of a breakpoint, but it is easy to determine whether or not a region contains a breakpoint. So rather than defining annotations in terms of precise breakpoint locations, we instead define them in terms of regions. For every region, we record the number of breakpoints that an expert expects in that region. These annotated regions can then be used to select an appropriate model, as shown in Figures 1 and 2.

We note that using databases of visual annotations is not a new idea, and has been used successfully for object recognition in photos and cell phenotype recognition in microscopy [17,18]. In array CGH analysis, some models can incorporate prior knowledge of locations of CNAs [19], but no models have been specifically designed to exploit visual breakpoint annotations.

Our second contribution is a protocol to estimate the breakpoint detection ability of the trained smoothing models on real data. In the Methods section, we propose to estimate the false positive and false negative rates of the trained models using cross-validation. This provides a quantitative criterion for deciding which smoothing algorithms are appropriate breakpoint detectors for which data.

The third contribution of this paper is a systematic, quantitative comparison of the accuracy of 17 common smoothing algorithms on a new database of 575 annotated neuroblastoma copy number profiles, which we give in the Results and discussion section. There are several publications which attempt to assess the accuracy of smoothing algorithms, and these methods fall into 2 categories: simulations and low-throughput experiments. GLAD, DNAcopy, and a hidden Markov model were compared by examining false positive and false negative rates for detection of a breakpoint at a known location in simulated data [20]. However, there is no way to verify if the assumptions of the simulation hold in a real data set, so the value of the comparison is limited. In another article, the accuracy of the CNVfinder algorithm was assessed using quantitative PCR [21]. But quantitative PCR is low-throughput and costly, so is not routinely done as a quality control. So in fact there are no previous studies that quantitatively compare breakpoint detection of smoothing models on real data. In this paper we propose to use annotated regions for quantifying smoothing model accuracy, and we make available 575 new annotated neuroblastoma copy number profiles as a benchmark for the community to test new algorithms on real data.

Several authors have recently proposed methods for so-called joint segmentation of multiple CGH profiles, under the hypothesis that each profile shares breakpoints in the exact same location [22,23]. These models are not useful in our setting, since we assume that breakpoints do not occur in the exact same locations across copy number profiles. Instead, we focus on learning a model that will accurately detect an unknown, different number of breakpoints in each copy number profile.

To summarize, this article describes a quantitative method for DNA copy number profile smoothing model selection. First, an expert examines scatterplots of the data, and encodes her interpretation of the breakpoint locations in a database of annotated regions. To repeat, the annotations represent an expert’s interpretation, not the biological truth in the tumor samples, which is unknown. We treat the annotated regions as a gold standard, and compare them to the breakpoints detected by 17 existing models. The best model for our expert is the one which maximizes agreement with the annotation database.

## Results and discussion

**Figure 3:**
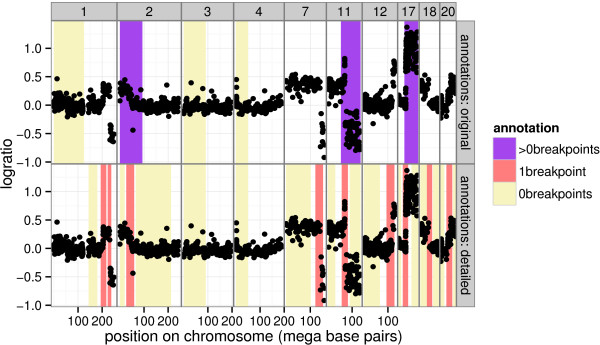
**Two annotation data sets of the same profile.** The annotated chromosomes of profile *i*=8, with the two sets of annotations shown in the two rows of plots. For the original annotations, the same 6 regions on each profile were systematically labeled as either 0breakpoints or >0breakpoints. For the detailed annotations, a GUI was used to draw regions anywhere on the profile, and 1breakpoint annotations were also used.

The 17 smoothing models described in the Algorithms: copy number profile smoothing models section were applied to 575 neuroblastoma copy number profiles, described in the Data: neuroblastoma copy number profiles section. After fitting the models, we used breakpoint annotations to quantify the accuracy of each model. We constructed 2 annotation databases based on 2 different experts’ interpretations of the same 575 profiles (Table 1). The “original” annotations were created by typing 0 or 1 in a 6-column spreadsheet after systematic inspection of the same 6 regions on each profile. The “detailed” annotations were constructed by using GUIs which allow zooming and direct annotation on the plotted profiles. The 2 annotation data sets are mostly consistent, but the detailed annotations provide more precise breakpoint locations (Figure 3).

**Table 1:** Counts of annotations in two annotation data sets of the same copy number profiles

	**Original**	**Detailed**
protocol	Systematic	Any
annotated profiles	575	575
annotated chromosomes	3418	3730
annotations	3418	4359
0breakpoints	2845	3395
1breakpoint	0	521
>0breakpoints	573	443

For the two annotation data sets (columns), we show the annotation protocol, counts of annotated profiles and chromosomes, and counts of annotations.

For both annotation databases, we calculated the global and local error curves *E*^global^(*λ*) and $E_{i}^{\text{local}}(\lambda )$, which quantify how many breakpoint annotations disagree with the model breakpoints. As shown in Figure 2 for the original set of annotations, the smoothness parameter *λ* is chosen by minimizing the error curves.

### Among global models, cghseg.k and pelt.n exhibit the smallest training error

The global model is defined as the smoothness parameter $\hat{\lambda }$ that minimizes the global error *E*^global^(*λ*), which is the total number of incorrect annotations over all profiles. Training error curves for cghseg.k, pelt.n, flsa.norm, and dnacopy.sd are shown in Figure 2. An ideal global model would have zero annotation error $E^{\text{global}}(\hat{\lambda })=0$ for some smoothness parameter $\hat{\lambda }$. However, none of the global models that we examined achieved zero training error in either of the two annotation databases. The best global models were the equivalent cghseg.k and pelt.n models, which achieved the minimum error of 2.2% and 6.1% in the original and detailed data sets.

**Figure 4:**
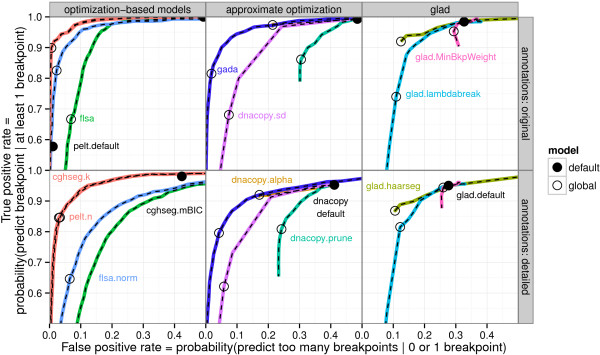
**ROC curves compare breakpoint detection of global models on two annotation data sets.** ROC curves for the training error with respect to the breakpoint annotation data are shown as colored lines. The curves are shown in 3 panels zoomed to the upper left region of ROC space to avoid visual clutter. Each curve is traced by plotting the error of a model as the degree of smoothness is varied, and an empty black circle shows the global model chosen by minimizing the error with respect to all annotations. Algorithms with no tuning parameters are shown as black dots. Note that some ROC curves appear incomplete since some segmentation algorithms are not flexible enough for the task of breakpoint detection, even though we ran each algorithm on a very large range of smoothness parameter values.

The ROC curves for the training error of the global models for each algorithm are traced in Figure 4. It is clear that the default parameters of each algorithm show relatively large false positive rates. The only exception is the pelt.default algorithm, which showed low false positive and true postive rates. The models chosen by maximizing agreement with the breakpoint annotation data also exhibit smaller false positive rates at the cost of smaller true positive rates. The ROC curves suggest that the equivalent cghseg.k and pelt.n models are the most discriminative for breakpoint detection in the neuroblastoma data.

### Among local models, cghseg.k and pelt.n exhibit the smallest training error

Since there is no global model that agrees with all of the annotations in either database, we fit local models with profile-specific smoothness parameters ${\hat{\lambda }}_{i}$. For every profile *i*, the local model is defined as the smoothness parameter ${\hat{\lambda }}_{i}$ that minimizes the local error $E_{i}^{\text{local}}(\lambda )$, the number of incorrect annotations on profile *i*. As shown in Figure 2, the local model fits the annotations at least as well as the global model: $E_{i}^{\text{local}}({\hat{\lambda }}_{i})\leq E_{i}^{\text{local}}(\hat{\lambda })$. However, the local model does not necessarily attain zero error. For example, Figure 2 shows that dnacopy.sd does not detect a breakpoint in profile *i*=362 even at the smallest parameter value, corresponding to the model with the most breakpoints.

**Figure 5:**
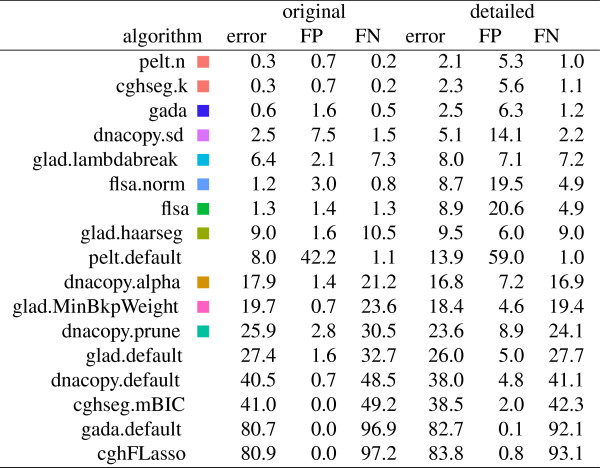
**Training error, false positive (FP) and false negative (FN) were calculated for all 17 algorithms applied to the original and the detailed annotation databases.** For each profile and algorithm, the smoothing parameter with minimal breakpoint annotation error was selected, and we report the mean training error across all profiles. Squares show the same colors as in the figures, and are absent for default models that have no smoothness parameters.

In Figure 5, we compare the fitting ability of the local models on the 2 annotation data sets. It clearly shows that some models are better than others for fitting the expert annotations of the neuroblastoma data. In particular, the equivalent cghseg.k and pelt.n local models show the best fit, with 0.3% and 2.1% error in the original and detailed data sets. Note that these are lower error rates than the global models, as expected.

But even if the local models are better at fitting the given breakpoint annotations, they do not generalize well to un-annotated breakpoints, as we show in the next section.

### Global models detect un-annotated breakpoints better than default models

**Figure 6:**
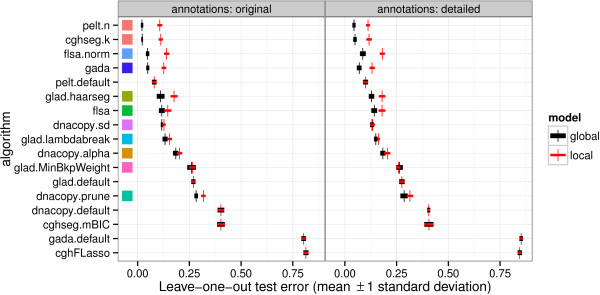
**Global models are better breakpoint detectors than local models.** Leave-one-out cross-validation over the annotions on each profile was used to compare breakpoint detection error of global and local models. The two panels show the two annotation data sets, and each row shows the performance of one of the models described in the Algorithms: copy number profile smoothing models section. After selecting the smoothness parameter *λ* by minimizing either the global or local annotation error, we plot the mean and standard deviation over 10 test sets. Each default model does not have a smoothness parameter, and shows equivalent local and global model error. Squares show the same colors as the other figures and tables, and are absent for default models that have no smoothness parameters.

Leave-one-out cross-validation was used to estimate the breakpoint detection of each model. Figure 6 shows the error rates of each model, across both annotation data sets. It is clear that the training procedure makes no difference for models pelt.default, glad.default, dnacopy.default, cghseg.mBIC, gada.default and cghFLasso, which are default models with no smoothness parameters. Each of these models is inferior to its respective global model in terms of breakpoint detection. The large error of these models suggest that the assumptions of their default parameter values do not hold in the neuroblastoma data set. More generally, these error rates suggest that smoothness parameter tuning is critically important to obtain an accurate smoothing of real copy number profiles.

**Figure 7:**
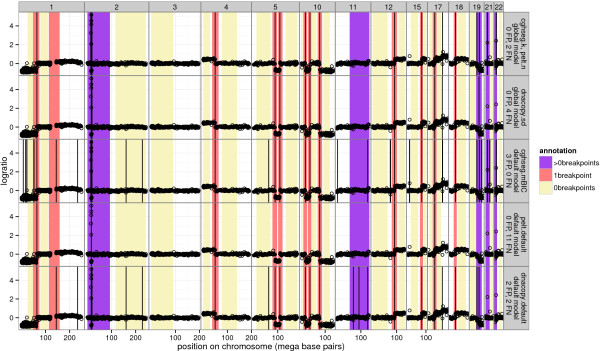
**Learned global models are better breakpoint detectors than default models.** Chromosomes with detailed annotations for neuroblastoma copy number profile *i*=207 are plotted in panels from left to right, and each row shows the breakpoints detected by a model as vertical black lines. The bottom 3 rows show default models trained using no annotations, and the top 2 rows show global models trained using annotations of other profiles. The equivalent cghseg.k and pelt.n global models show only 2 annotation errors compared to 3 and 11 in the corresponding default models. Furthermore, the equivalent cghseg.k and pelt.n global models perform better than dnacopy.sd, which shows 4 annotation errors.

To show an example of how the learned models outperform default models, Figure 7 shows one representative profile with many breakpoints. Note that the models were trained on other profiles, so the shown annotations can be used for model evaluation. For this profile, dnacopy.default shows 2 false positives and 2 false negatives, and dnacopy.sd shows no improvement with 4 false negatives. The pelt.default and cghseg.mBIC show 11 and 3 annotation errors, respectively. The cghseg.k and pelt.n global models show only 2 annotation errors, demonstrating the usefulness of annotation-based model training.

In addition, Additional file 1: Figure S1-S5 compares these default and global models. Again, the global models were learned on other profiles, so the shown annotations can be used for model evaluation.

### Global models detect un-annotated breakpoints better than local models

The leave-one-out cross-validation results in Figure 6 also allow comparison of global and local models. For dnacopy.prune, glad.MinBkpWeight, glad.lambdabreak, dnacopy.sd and flsa, there is little difference between the local and global training procedures. For models flsa.norm, gada, pelt.n, and cghseg.k, there is a clear advantage for the global models which share information between profiles. The equivalent cghseg.k and pelt.n models show the minimal test error of only 2.1% and 4.4% in the original and detailed data sets.

### Only a few profiles need to be annotated for a good global model

To estimate the generalization error of a global model trained on a relatively small training set of *t* annotated profiles, we applied ⌊*n*/*t*⌋-fold cross-validation to the *n*=575 profiles.

**Figure 8:**
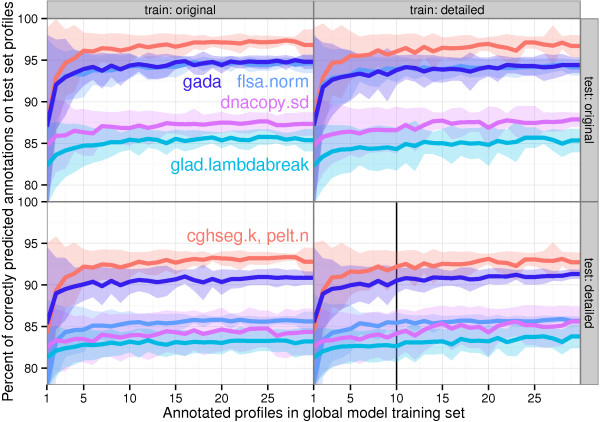
**Test accuracy increases to a model-specific limit as number of training profiles increases.** Cross-validation was used to estimate the generalization ability of the global models with different sized training sets. Panels from left to right specify the annotations that were used to train, and panels from top to bottom specify the annotations that were used to test. Note that the results change very little even when training on one data set and testing on another. For each training set size *t*, the profiles were partitioned into training sets of approximately size *t*, then were evaluated using the annotations from all the other profiles. Results on these data indicate increasing accuracy (lines) and decreasing standard deviation (shaded bands) as the training set increases. The accuracy of each model quickly attains its maximum, after only about *t*=10 profiles. In Figure 9, we show the results for all algorithms when trained on *t*=10 profiles from the detailed data set, and tested on the other profiles in the detailed data set (vertical black line).

For several training set sizes *t*, we plot the accuracy of the cghseg.k, pelt.n, gada, flsa.norm, dnacopy.sd and glad.lambdabreak models in Figure 8. It shows that adding more annotations to the training set increases the breakpoint detection accuracy in general, but at a diminishing rate. Each model quickly attains its specific maximum, after only about *t*=10 training profiles.

**Figure 9:**
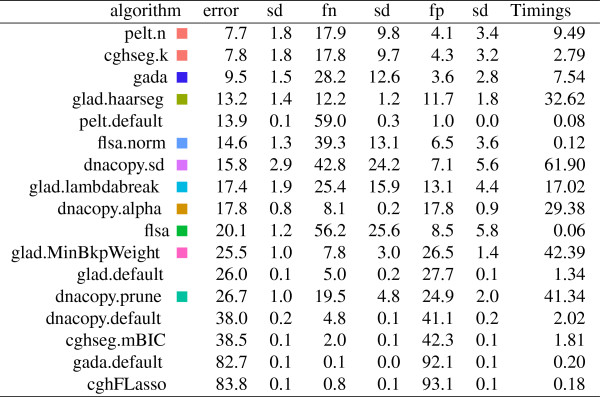
**The *n/t*-fold cross-validation protocol was used to estimate error, false positive (fp), and false negative (fn) rates in the detailed annotations of the *n *= 575 profiles.** Squares show the same colors as in the figures, and are absent for default models that have no smoothness parameters. The smoothness parameter was chosen using annotations from approximately *t*=10 profiles, and mean and standard deviation (sd) of test error over ⌊*n*/*t*⌋=57 folds are shown as percents. The Timings column shows the median number of seconds to fit the sequence of smoothing models for a single profile.

In Figure 9, we used ⌊*n*/*t*⌋-fold cross-validation in the detailed annotations to estimate the error rates of all 17 models trained using only *t*=10 profiles.

The equivalent cghseg.k and pelt.n models show the best performance on these data, with an estimated breakpoint detection error of 7.7*%*.

### Global models generalize across annotators

We assessed the extent to which the annotator affects the results by comparing models trained on one data set and tested on the other. Figure 8 shows that test error changes very little between models trained on one data set or the other. This demonstrates that global models generalize very well across annotators.

### Timing PELT and cghseg

The PELT and cghseg models use different algorithms to calculate the same segmentation, which showed the best breakpoint detection performance in every comparison. But they are slightly different in terms of speed, as we show in Figure 9.

When comparing the global models, cghseg.k is somewhat faster than pelt.n. For cghseg.k, pruned dynamic programming is used to calculate the best segmentation *μ*^*k*^ for k∈{1,…,20} segments, which is the slow step. Then, we calculate the best segmentation for $\lambda \in \{\lambda_{1},\ldots ,\lambda_{100}\}$, based on the stored *μ*^*k*^ values. In contrast, the Pruned Exact Linear Time algorithm must be run for each $\lambda \in \{\lambda_{1},\ldots ,\lambda_{100}\}$, and there is no information shared between *λ* values.

Timing the PELT and cghseg default models without tuning parameters shows the opposite trend. In particular, the default cghseg.mBIC method is slower than the pelt.default method. This makes sense since cghseg must first calculate the best segmentation *μ*^*k*^ for several *k*, then use the mBIC criterion to choose among them. In contrast, the PELT algorithm recovers just the *μ*^*k*^ which corresponds to the Schwarz Information Criterion penalty constant *β*= log*d*. So if you want to use a particular penalty constant *β* instead of the annotation-guided approach we suggest in this article, the default PELT method offers a modest speedup over cghseg.

### Annotation-based modeling is feasible for high-density microarrays

**Figure 10:**
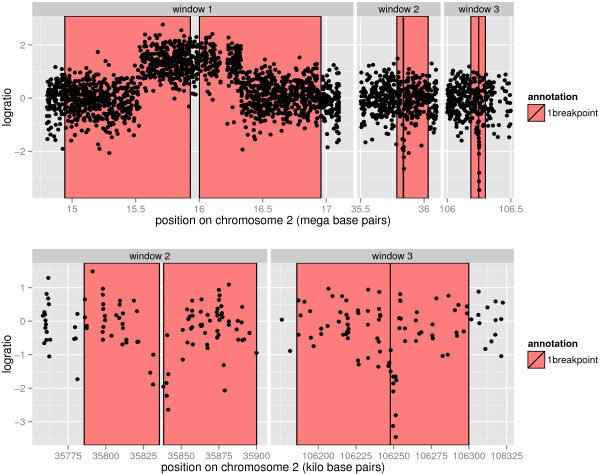
**Zooming allows creation of small annotated regions on high-density profiles. Top**: three regions of chromosome 2 from a high-density Affymetrix SNP6 array are shown along with some breakpoint annotations. There are almost 2 million probes on the array, 153663 probes on chromosome 2, and 2549 probes shown in these 3 windows. **Bottom**: zoom to show detail of windows 2 and 3. Annotations can be used to ensure that a smoothing model accurately recovers breakpoints around small ≈10kb alterations such as those shown in windows 2 and 3.

Although not the main focus of this paper, we have already started to apply annotation-based modeling to high-density microarrays. For example, Figure 10 shows part of chromosome 2 from an Affymetrix SNP6 microarray. This microarray offers almost 2 million probes, and we show annotated breakpoints around 3 CNAs from ≈1Mb to ≈10 kb. As long as there is GUI software that supports zooming and annotation, it is feasible to apply annotation-based modeling.

## Conclusions

We proposed to train breakpoint detection models using annotations determined by visual inspection of the copy number profiles. We have demonstrated that this approach allows quantitative comparison of smoothing models on a new data set of 575 neuroblastoma copy number profiles. These data provide the first set of annotations that can be used for benchmarking the breakpoint detection ability of future algorithms. Our annotation-based approach is quite useful in practice on real data, since it provides a quantitative criterion for choosing the model and its smoothing parameter.

One possible criticism of annotation-based model selection is the time required to create the annotations. However, using the GUIs that we have developed, it takes only a few minutes to annotate the breakpoints in a profile. This is a relatively small investment compared to the time required to write the code for data analysis, which is typically on the order of days or weeks. In addition, in the neuroblastoma data, we observed that annotating only about 10 of 575 profiles was sufficient to learn a smoothness parameter that achieves the model-specific optimal breakpoint detection. More generally, our results suggest that after obtaining a moderately sized database of annotations, data analysis time is better spent designing and testing better models. Additionally, the learned models generalized very well between annotators. So breakpoint annotations are a feasible approach for finding an accurate model and smoothing parameter for real copy number profiles.

We compared local models for single profiles with global models selected using annotations from several profiles. We observed that local models fit the given annotations better, but global models generalize better to un-annotated regions. In contrast with our results, it has been claimed that local models should be better in some sense: “it is clear that the advantages of selecting individual-specific *λ* values outweigh the benefit of selecting constant *λ* values that maximize overall performance” [15]. However, they did not demonstrate this claim explicitly, and one of the contributions of this work is to show that global models generalize better than local models, according to our leave-one-out estimates.

It will be interesting to apply annotation-based model training to other algorithms and data sets. In both annotation data sets we analyzed, cghseg.k and pelt.n showed the best breakpoint detection, but another model may be selected for other data.

Our results indicate that even the best models have non-zero training and testing breakpoint detection error, which could be improved. To make a model that perfectly fits the training annotations, a dynamic programming algorithm called SegAnnot was proposed to recover the most likely breakpoints that are consistent with the annotation data [24]. The test error of the cghseg model can be lowered by choosing chromosome-specific *λ* parameter values as a function of features such as variance and the number of points sampled [25]. Developing a model that further lowers the test error remains an interesting direction of future research.

We have solved the problem of smoothness parameter selection using breakpoint annotations, but the question of detecting CNAs remains. By constructing a database of annotated regions of CNAs, we could use a similar approach to train models that detect CNAs. Annotations could be actual copy number (0,1,2,3,…) or some simplification (loss, normal, gain). We will be interested in developing joint breakpoint detection and copy number calling models that directly use these annotation data as constraints or as part of the model likelihood.

## Methods

### GUIs for annotating copy number profiles

Assume that we have *n* DNA copy number profiles, and we would like to accurately detect their breakpoints. The first step of annotation-based modeling is to plot the data, visually identify breakpoints, and save these regions to an annotation database. We created 2 annotation graphical user interfaces (GUIs) for this purpose: a Python program for low-density profiles called annotate_breakpoints.py, and a web site for larger profiles called SegAnnDB.

We used Tkinter in Python’s standard library to write annotate_breakpoints.py, a cross-platform GUI for annotating low-density DNA copy number profiles. The annotator loads several profiles from a CSV file, plots the data, and allows annotated regions to be drawn on the plot and saved to a CSV file for later analysis. The annotator does not support zooming so is not suitable for annotating high-density profiles. It is available in the annotate_regions package on the Python Package Index:

http://pypi.python.org/pypi/annotate_regions

SegAnnDB is a web site that can be used to annotate low to high-density copy number profiles. After copy number data in bedGraph format is uploaded, the site uses D3 to show plots which can be annotated [26]. The annotations can then be downloaded for later analysis. As shown in Figure 10, the plots can be zoomed for detailed annotation of high-density copy number profiles. The free/open-source software that runs the web site can be downloaded from the breakpoints project on INRIA GForge:

https://gforge.inria.fr/scm/viewvc.php/webapp/?root=breakpoints

### Definition of breakpoints in smoothing models

For each profile i∈{1,…,n}, we observe $d_{i}\in \mathbb{N}$ noisy logratio measurements $y_{i}\in {\mathbb{R}}^{d_{i}}$. Assume that we have a model with smoothness parameter *λ* that takes the vector of logratios *y*_*i*_ and outputs a smoothed signal ${ŷ}_{i}^{\lambda }\in {\mathbb{R}}^{d_{i}}$. For simplicity of notation, let ${\hat{x}}^{\lambda }\in {\mathbb{R}}^{m}$ be the smoothed signal sampled at positions $p_{1}\leq \cdots \leq p_{m}$ on one chromosome of one profile. We define the breakpoints predicted by this model as the set of positions where there are jumps in the smoothed signal:

(1) $${\hat{b}}^{\lambda }=\left\{\left(p_{j}+p_{j+1})/2|{\hat{x}}_{j}^{\lambda }\neq {\hat{x}}_{j+1}^{\lambda },\;\forall j\in \{1,\ldots ,m-1\right\}\right\}$$

Note that this set is drawn using vertical black lines in Figures 1 and 7.

### Definition of the annotation error

For every profile and chromosome, we judge the accuracy of the predicted set of breakpoints ${\hat{b}}^{\lambda }$ using a set of visually-determined regions and corresponding annotations. Every annotation $a=\;[\;\underline{a},\bar{a}]$ is an interval that specifies the expected number of changes in the corresponding region *r*. For example, we defined 3 types of annotations: *a*= [ 0,0] for 0breakpoints annotations, *a*= [ 1,1] for 1breakpoint annotations, and *a*= [ 1,*∞*) for >0breakpoints annotations. A region is an interval of base pairs on the chromosome that corresponds to ${\hat{b}}^{\lambda }$, for example *r*= [ 1000000,2000000].

The false positives (FP) and false negatives (FN) are calculated by comparing the estimated number of changes in each region $\left|{\hat{b}}^{\lambda }\cap r\right|$ to the annotated number of changes *a* using the zero-one loss:

(2) $$\text{FP}({\hat{b}}^{\lambda },r,a)=\left\{\begin{array}{ll} 1 & \text{if}|{\hat{b}}^{\lambda }\cap r|>\bar{a},\\ 0 & \text{otherwise.} \end{array}\right)$$

(3) $$\text{FN}({\hat{b}}^{\lambda },r,a)=\left\{\begin{array}{ll} 1 & \text{if}|{\hat{b}}^{\lambda }\cap r|<\underline{a},\\ 0 & \text{otherwise.} \end{array}\right)$$

Note that >0breakpoints annotations can never have false positives $\text{FP}({\hat{b}}^{\lambda },r,[1,\infty ))=0$ and 0breakpoints annotations can never have false negatives $\text{FN}({\hat{b}}^{\lambda },r,[\;0,0])=0$. The annotation error is defined as the sum of false positives and false negatives:

(4) $$e({\hat{b}}^{\lambda },r,a)=\text{FP}({\hat{b}}^{\lambda },r,a)+\text{FN}({\hat{b}}^{\lambda },r,a)=\left\{\begin{array}{ll} 0 & \text{if}|{\hat{b}}^{\lambda }\cap r|\in a\\ 1 & \text{otherwise.} \end{array}\right)$$

Note that this loss function gives the same weight to false positives and false negatives. Re-weighting schemes could be used, but uniform weighting is justified in the data we analyzed since each annotation took approximately the same amount of time to create.

### Definitions of error and ROC curves

For each profile *i*, we define the local error as the total annotation error over all annotated regions:

(5) $$E_{i}^{\text{local}}(\lambda )=\underset{({\hat{b}}^{\lambda },r,a)\text{on profile}\;i}{\sum }e({\hat{b}}^{\lambda },r,a).$$

We define the global error as the total annotation error over all profiles:

(6) $$E^{\text{global}}(\lambda )=\overset{n}{\underset{i=1}{\sum }}E_{i}^{\text{local}}(\lambda ).$$

For a given algorithm, a ROC curve is drawn by plotting the true positive rate TPR(*λ*) against the false positive rate FPR(*λ*) for all values of *λ*. For one annotation, the true positive indicator function is

(7) $$\text{TP}({\hat{b}}^{\lambda },r,a)=\left\{\begin{array}{ll} 1 & \text{if}|{\hat{b}}^{\lambda }\cap r|\geq \underline{a}\\ 0 & \text{otherwise.} \end{array}\right)$$

To define the true positive rate, we first define the set of positive annotations $\left({\hat{B}}_{+}^{\lambda },R_{+},A_{+}\right)$ as all the annotations *a*∈*A*_+_ such that there is at least one breakpoint $\underline{a}\geq 1$. The true positive rate over all positive annotations is

(8) $$\text{TPR}(\lambda )=\frac{1}{\left|A_{+}\right|}\underset{\left({\hat{b}}^{\lambda },r,a)\in ({\hat{B}}_{+}^{\lambda },R_{+},A_{+}\right)}{\sum }\text{TP}({\hat{b}}^{\lambda },r,a).$$

To define the false positive rate, we first define the set of negative annotations $\left({\hat{B}}_{-}^{\lambda },R_{-},A_{-}\right)$ as all the annotations that could possibly have a false positive: $\bar{a}<\infty$. Then the false positive rate over all negative annotations is

(9) $$\text{FPR}(\lambda )=\frac{1}{\left|A_{-}\right|}\underset{\left({\hat{b}}^{\lambda },r,a)\in ({\hat{B}}_{-}^{\lambda },R_{-},A_{-}\right)}{\sum }\text{FP}({\hat{b}}^{\lambda },r,a).$$

### Selecting the optimal degree of smoothness

We assume that *λ* is a tuning parameter that is monotonic in the number of breakpoints, which is the case for the models considered in this paper. Fix a set of smoothing parameters *λ*∈*Λ*, and run the smoothing algorithm with each of these parameters. Intuitively, we should select the value of *λ* that maximizes agreement with annotation data. For global models, we minimize the global error, and there is usually one best value:

(10) $$\hat{\lambda }=\underset{\lambda \in \Lambda }{\arg\;\min}\;E^{\text{global}}(\lambda ).$$

For the local model for profile *i*, we want to minimize the local error:

(11) $${\hat{\lambda }}_{i}=\underset{\lambda \in \Lambda }{\arg\;\min}\;E_{i}^{\text{local}}(\lambda ).$$

Since the training set consists of only the annotations of one profile *i*, there may be several smoothing parameters *λ* that minimize the error. We propose to choose between models that achieve the minimum error based on the shape of the error curve, and these cases are illustrated in Figure 2. 

1. When the minimum error is achieved in a range of intermediate parameter values, we select a value in the middle. This occurs in the local error curves shown for flsa.norm and cghseg.k.

2. When the minimum is attained by the model with the most breakpoints, we select the model with the fewest breakpoints that has the same error. This attempts to minimize the false positive rate. This occurs for profile *i*=375 with the dnacopy.sd model.

3. When the minimum is attained by the model with the fewest breakpoints, we select the model with the most breakpoints that has the same error. This attempts to minimize the false negative rate, and occurs for profile *i*=362 with the dnacopy.sd model.

More complicated smoothing parameter estimators could be defined, but for simplicity in this article we explore only the global $\hat{\lambda }$ and local ${\hat{\lambda }}_{i}$ models.

### Leave-one-out cross-validation for comparing local and global models

To compare the breakpoint detection performance of local and global models, we propose to leave one annotation per profile aside as a test set. The input parameter *V* is the number of times the procedure is repeated. In our analysis we took *V*=10 repetitions. For each repetition, 

1. On each profile, randomly pick one annotated region and set it aside as a test set.

2. Using all the other annotations as a training set, select the best *λ* using the protocol described in Section “Selecting the optimal degree of smoothness” For local models we learn a profile-specific ${\hat{\lambda }}_{i}$ that minimizes $E_{i}^{\text{local}}$, and for global models we learn a global $\hat{\lambda }$ that minimizes *E*^global^.

3. To estimate how the model generalizes, count the errors of the learned model on the test regions.

The final estimate of model error shown in Figure 6 is the average error over all *V* repetitions.

### ⌊*n*/*t*⌋-fold cross-validation to estimate error on un-annotated profiles

Since the annotation process is time-consuming, we are interested in training an accurate breakpoint detector with as few annotations as possible. Thus we would like to answer the following question: how many profiles *t* do I need to annotate before I get a global model that will generalize well to all the other profiles?

To answer this question, we estimate the error of a global model trained on the annotations from *t* profiles using cross-validation. We divide the set of *n* annotated profiles into exactly ⌊*n*/*t*⌋ folds, each with approximately *t* profiles. For each fold, we consider its annotations a training set for a global model, and combine the other folds as a test set to quantify the model error. The final estimate of generalization error is then the average model error over all folds.

### Data: neuroblastoma copy number profiles

We analyzed a new data set of *n*=575 copy number profiles from aCGH microarray experiments on neuroblastoma tumors taken from patients at diagnosis. The microarrays were produced using various technologies, so do not all have the same probes. The number of probes per microarray varies from 1719 to 71340, and the median spacing between probes varies from 40 Kb to 1.2 Mb. In this article we analyzed the normalized logratio measurements of these microarrays, which we have made available as neuroblastoma$profiles in R package neuroblastoma on CRAN.

Two different expert annotations were used to construct 2 annotation databases based on these profiles (Table 1). The 2 annotation data sets are mostly consistent, but the detailed annotations provide more precise breakpoint locations (Figure 3).

The “original” annotations were created using the “Systematic” protocol. First, a set of 6 genomic regions was chosen. Then, each of these regions was inspected on scatterplots of each profile. Breakpoint annotations were recorded by typing 0 or 1 in spreadsheet with one row for each of the 575 profiles and one column for each of the 6 regions. Entries with 0 were 0breakpoints annotations *a*= [ 0,0] and entries with 1 were >0breakpoints annotations *a*= [ 1,*∞*). These annotations are available as neuroblastoma$annotations in R package neuroblastoma on CRAN.

The “detailed” annotations were constructed using the “Any” protocol. The data were shown as scatterplots in a graphical user interface (GUI) that allows zooming and direct annotation on the plotted profiles. Annotators were asked to label any regions for which they were sure of the annotation. These annotations are available as neuroblastomaDetailed in R package bams on CRAN.

### Algorithms: copy number profile smoothing models

We considered smoothing models from the bioinformatics literature with free software implementations available as R packages on CRAN, R-Forge, or Bioconductor [27-29]. For each algorithm, we considered three types of training for the smoothness parameter *λ*: 

• **Default models** can be used when functions give default parameter values, or do not have smoothness parameters that vary the number of breakpoints.

• **Local models** choose a smoothness parameter that maximizes agreement with the annotations from a single profile.

• **Global models** choose a smoothness parameter that maximizes agreement with the entire database of training annotations.

In the following paragraphs, we discuss the precise meaning of the smoothness parameter *λ* in each of the algorithms. The code that standardizes the outputs of these models can be found in the list of functions smoothers in R package bams on CRAN. For some algorithms (GADA, GLAD, DNAcopy) the smoothing ${ŷ}^{\lambda }\in {\mathbb{R}}^{d}$ is defined for an entire profile $y\in {\mathbb{R}}^{d}$, but in others (cghseg, pelt, flsa) the smoothing ${\hat{x}}^{\lambda }\in {\mathbb{R}}^{m}$ is defined in terms of probes on a single chromosome $x\in {\mathbb{R}}^{m}$. Note that to decrease computation time, the model fitting may be trivially parallelized for profiles, algorithms, and smoothing parameter values.

We used version 1.0 of the gada package from R-Forge to calculate a sparse Bayesian learning model [11]. We varied the degree of smoothness by adjusting the T parameter of the BackwardElimination function, and for the gada.default model, we did not use the BackwardElimination function.

We used version 1.3 of the flsa package from CRAN to calculate the Fused Lasso Signal Approximator [10]. The FLSA solves the following optimization problem for each chromosome:

(12) $$\begin{array}{c} \;{\hat{x}}^{\lambda }\;=\underset{\mu \in {\mathbb{R}}^{m}}{\arg\;\min}\frac{1}{2}\overset{m}{\underset{i=1}{\sum }}{\left(x_{i}-\mu_{i}\right)}^{2}\;+\lambda_{1}\overset{m}{\underset{i=1}{\sum }}|\mu_{i}|\;+\lambda_{2}\overset{m-1}{\underset{i=1}{\sum }}|\mu_{i}\;-\;\mu_{i+1}|. \end{array}$$

We define a grid of values $\lambda \in \{10^{-5},\ldots ,10^{12}\}$, take *λ*_1_=0, and consider the following parameterizations for *λ*_2_: 

• flsa: *λ*_2_=*λ*.

• flsa.norm: *λ*_2_=*λm*×10^6^/*l* where *m* is the number of points and *l* is the length of the chromosome in base pairs.

We used version 1.18.0 of the DNAcopy package from Bioconductor to fit a circular binary segmentation model [7]. We varied the degree of smoothness by adjusting the undo.SD, undo.prune, and alpha parameters of the segment function. However, the dnacopy.prune algorithm was too slow ( >24 hours) for some of the profiles with many data points, so these profiles were excluded from the analysis of dnacopy.prune.

We used version 0.2.1 of the cghFLasso package from CRAN, which implements a default fused lasso method [8], but does not provide any smoothness parameters for breakpoint detection.

We used version 2.0.0 of the GLAD package from Bioconductor to fit the GLAD adaptive weights smoothing model [5]. We varied the degree of smoothness by adjusting the lambdabreak and MinBkpWeight parameters of the daglad function. For the glad.haarseg model, we used the smoothfunc=~haarseg~ option and varied the breaksFdrQ parameter to fit a wavelet smoothing model [9].

To fit a Gaussian maximum-likelihood piecewise constant smoothing model [6], we used pruned dynamic programming as implemented in version 0.1 of the cghseg package from R-Forge [30]. For the default cghseg.mBIC model, we used the modified Bayesian information criterion [14], which has no smoothness parameter, and is implemented in the uniseg function of the cghseg package. For the cghseg.k model, we used the segmeanCO function with kmax=20 to obtain the maximum-likelihood piecewise constant smoothing model $\mu^{k}\in {\mathbb{R}}^{m}$ for k∈{1,…,20} segments. Lavielle suggested penalizing *k* breakpoints in a signal sampled at *m* points using *λk*, and varying *λ* as a tuning parameter [13]. We implemented this model selection criterion as the cghseg.k model, for which we define the optimal number of segments

(13) $$\begin{array}{c} k^{*}(\lambda )=\underset{k\in \{1,\ldots ,20\}}{\arg\;\min}\mathrm{\lambda k}+\frac{1}{m}\overset{m}{\underset{i=1}{\sum }}{\left(x_{i}-\mu_{i}^{k}\right)}^{2}, \end{array}$$

and the optimal smoothing

$${\hat{x}}^{\lambda }=\mu^{k^{*}(\lambda )}.$$

We used the cpt.mean function in version 1.0.4 of the changepoint package from CRAN to fit a penalized maximum likelihood model using a Pruned Exact Linear Time (PELT) algorithm [12]. PELT defines *μ*^*k*^ in the same way as cghseg, but defines the optimal number of segments as

(14) $$k^{*}(\beta )=\underset{k\in \{1,\ldots ,m\}}{\arg\;\min}\beta (k-1)+\overset{m}{\underset{i=1}{\sum }}{\left(x_{i}-\mu_{i}^{k}\right)}^{2}.$$

For the pelt.default model, we used the default settings which specify penalty=~SIC~ for the Schwarz or Bayesian Information Criterion, meaning *β*= log*m*. For the pelt.n model, we specified penalty=~Manual~ which means that the value parameter is used as *β*, and the cpt.mean function returns $\mu^{k^{*}(\beta )}$. We defined the same grid of *λ* values that we used for cghseg.k, and let *β*=*λd*. Note that this model is mathematically equivalent to cghseg.k, but shows small differences in the results, since there are rounding errors when specifying the penalty cpt.mean(value=sprintf(~n*%f~,lambda)) for pelt.n.

### Ethical approval

This study was authorized by the decision of the ethics comitee “Comité de Protection des Personnes Sud-Est IV”, reference L07-95 and L12-171.

## Competing interests

The authors declare that they have no competing interests.

## Authors’ contributions

OD, GS, IJ-L, and JC designed and executed the experiments. TDH, VB, GS, and IJ-L created the annotation databases. TDH, FB and J-PV conceived the statistical model selection framework. TDH wrote the code and manuscript. All authors read and approved the final manuscript.

## Supplementary Material

Additional file 1 **Supplementary figures.** Breakpoint detection performance of 5 models on 5 neuroblastoma copy number profiles. Global models were trained on other profiles, so the shown annotations were used to quantify model accuracy. Click here for file
