# Supplementary material for: Learning smoothing models of copy number profiles using breakpoint annotations
Source: BMC Bioinformatics. 2013 May 22;14:164. doi: 10.1186/1471-2105-14-164 (PMC3712326; doi:10.1186/1471-2105-14-164)
Supplement: Additional file 1 — Supplementary figures. Breakpoint detection performance of 5 models on 5 neuroblastoma copy number profiles. Global models were trained on other profiles, so the shown annotations were used to quantify model accuracy. [file 1471-2105-14-164-S1.pdf]

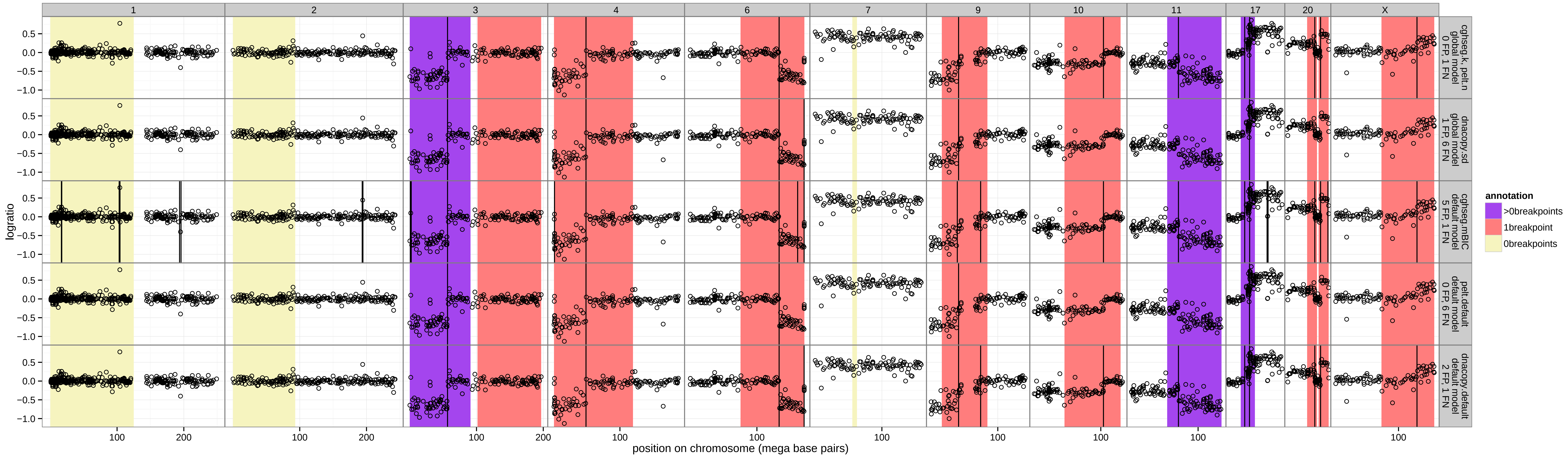

Figure S1. profile 18 This BAC array has approximately 1 probe per megabase, and shows many detailed breakpoint annotations. Global models were trained on other profiles, so the shown annotations were used to quantify model accuracy. In this case the dnacopy.sd global model has more annotations than the dnacopy.default model. The cghseg.k and pelt.n global models show only 1 false negative, so are more accurate breakpoint detectors than their default models. The "false negative" on chromosome 3 seems to be an incorrect annotation.

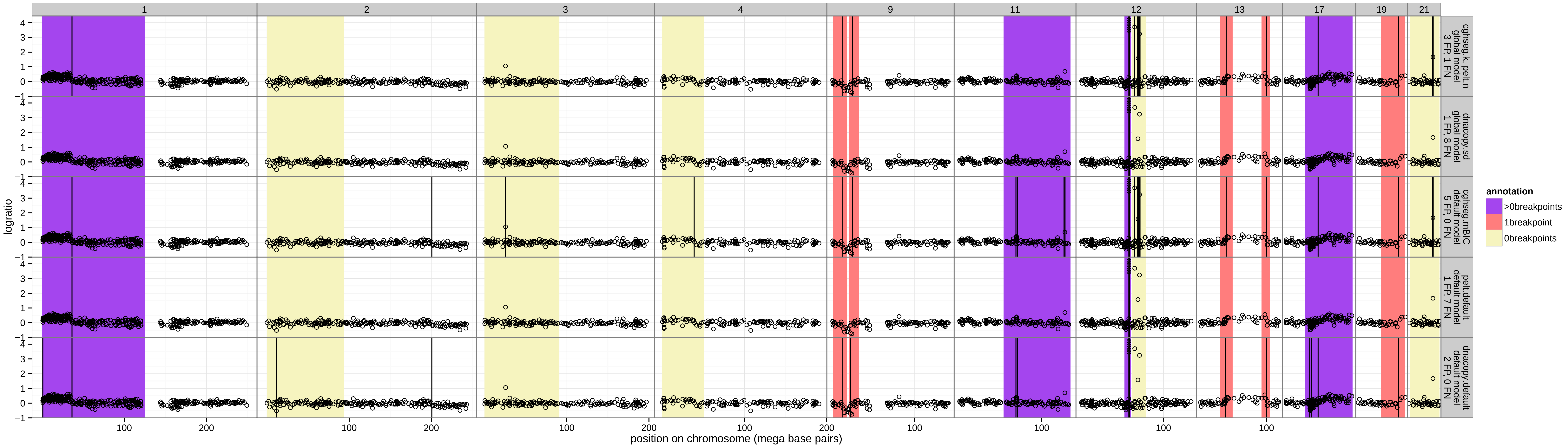

Figure S2. profile 22 This BAC array has approximately 1 probe per megabase, and shows many detailed breakpoint annotations. Global models were trained on other profiles, so the shown annotations were used to quantify model accuracy. This is one of the rare profiles for which dnacopy.default shows the lowest annotation error.

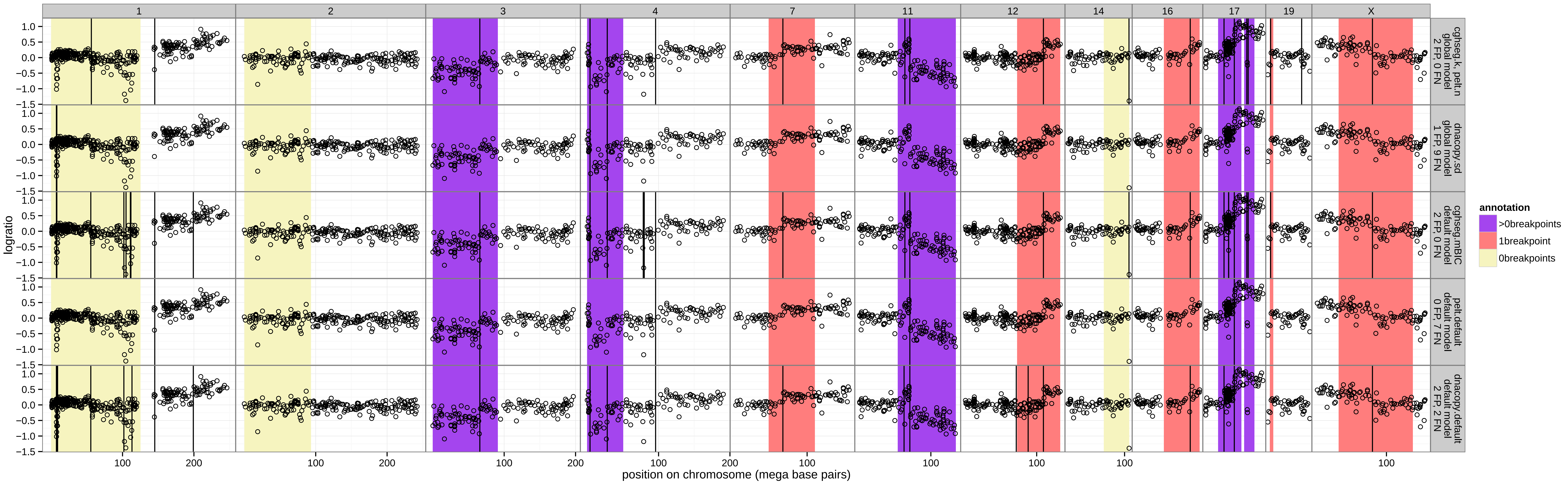

Figure S3. profile 123 This BAC array has approximately 1 probe per megabase, and shows many detailed breakpoint annotations. Global models were trained on other profiles, so the shown annotations were used to quantify model accuracy. The best models for this profile achieve 2 annotation errors: cghseg.k, pelt.n, and cghseg.mBIC. In the 0breakpoints region on chromosome 1, The cghseg.k algorithm detects 1 breakpoint and cghseg.mBIC detects 7.

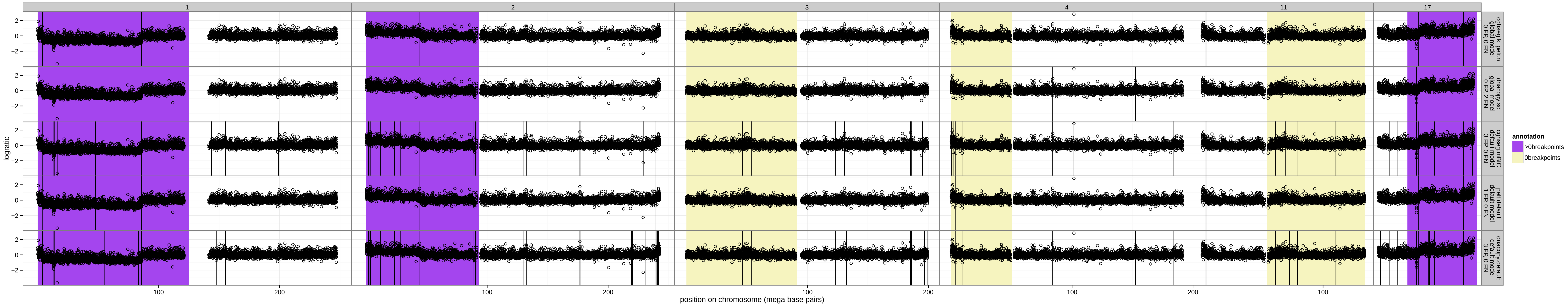

Figure S4. profile 546 This Nimblegen array has a probe every 40kb and shows 3 positive and 3 negative annotations. Global models were trained on other profiles, so the shown annotations were used to quantify model accuracy. Default models tend to detect false positives in this noisy profile. The learned global models in the top two rows show fewer annotation errors than the respective default models in the bottom 3 rows.

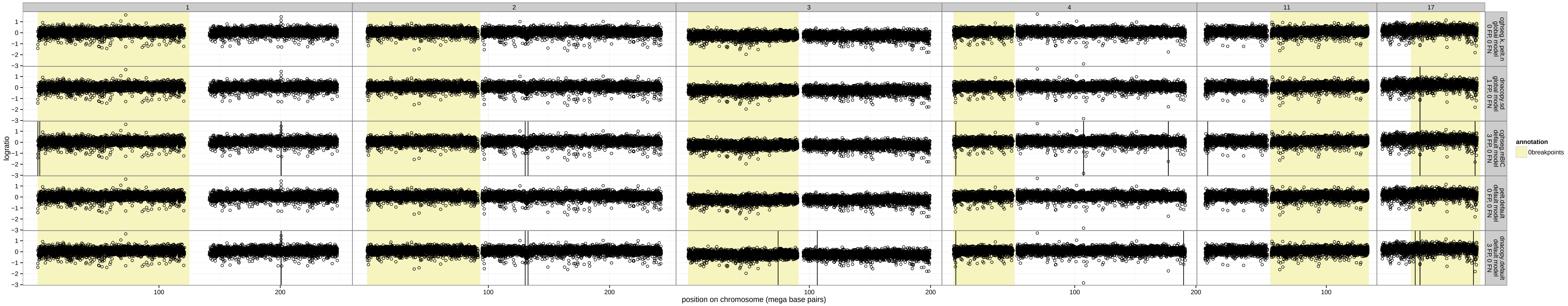

Figure S5 - profile 229 This Nimblegen array has a probe every 40kb and shows only negative annotations. Global models were trained on other profiles, so the shown annotations were used to quantify model accuracy. The learned global models in the top two rows show fewer false positives than the default models in the bottom 3 rows.
